# Development and Characterization of Water-Based Porous Calcium Phosphate Bone Cements for Peri-Implant Regeneration: An In Situ Study

**DOI:** 10.3390/biomimetics11080551

**Published:** 2026-08-03

**Authors:** Qiuju Wei, Nima Farshidfar, Anton Sculean, Mia Rakic

**Affiliations:** 1 Department of Periodontology, School of Dental Medicine, University of Bern, 3010 Bern, Switzerland; qiuju.wei@unibe.ch (Q.W.); anton.sculean@unibe.ch (A.S.); 2 Graduate School for Cellular and Biomedical Sciences, University of Bern, 3012 Bern, Switzerland

**Keywords:** bone adhesives, calcium phosphate, bone cements, dental implants, bone regeneration

## Abstract

Background: Calcium phosphate cements (CPCs) are excellent biomaterials for peri-implant bone regeneration but suffer from slow resorption. This study evaluated whether adding carbonate salts improves the in situ porosity and resorption rate of customized CPCs. Methods: The control group comprised α-tricalcium phosphate (α-TCP) and phosphoserine (3:1 weight-to-weight ratio). The test group incorporated 3 wt.% anhydrous sodium carbonate (Na_2_CO_3_) into the powder. Both were hydrated with water at a liquid-to-powder ratio of 300 μL:1 g. Characterization included micro-computed tomography (μCT), scanning electron microscopy (SEM), Fourier Transform Infrared Spectroscopy with Attenuated Total Reflection (FTIR-ATR), removal torque tests, compression modulus tests, and hardness tests. Results: μCT and SEM confirmed higher porosity and uniform crystal plates in the test group compared to the dense control group. FTIR-ATR spectra showed a distinct CO_2_ peak at 2349 cm^−1^ for the test group, confirming gas entrapment. Mechanically, the control group significantly outperformed the test group in removal torque (71.58 ± 5.56 N/cm vs. 41.37 ± 4.54 N/cm) and compression modulus (1248.01 ± 278.21 MPa vs. 195.42 ± 29.55 MPa). Hardness tests showed increased brittleness in the test group (15.31 ± 1.63 vs. 2.01 ± 1.58). Conclusions: Incorporating Na_2_CO_3_ successfully induced in situ porosity via gas release but significantly compromised mechanical strength. Further optimization is required to balance porosity and mechanical integrity.

## 1. Introduction

Implant stability is the ultimate prerequisite for successful dental implant therapy [1,2]. However, maintaining this stability is a continuous challenge, beginning at placement, progressing through changes in bone metabolism during osseointegration and functional loading, and facing risks from peri-implantitis [3]. For these reasons, bone substitutes are commonly used to provide support to the implant while serving as space holders and scaffolds for new bone ingrowth [4,5]. However, peri-implant bone regeneration poses a formidable challenge due to unique biological specificities and requirements, which is best depicted by the fact that the regeneration rate achieved with available bone grafting materials in periodontology rarely exceeds 50% [6,7]. This limitation is particularly critical in implant dentistry, given the lack of a periodontal ligament, which reduces the initial amortization capacity of dental implants and increases their susceptibility to biomechanical trauma. In brief, subjecting suboptimally stabilized dental implants to even physiological occlusal/masticatory loads induces bone resorption via osteocyte mechanosensing [8,9].

A major factor specific to dental implants is the macro- and micro-roughness of the implant surface, which complicates the adhesion of conventional bone substitute particles and compromises the quality of defect filling [10]. This limitation has stimulated a series of research into injectable calcium phosphate cements (CPCs) due to their unique ability to facilitate tissue adhesion and provide immediate structural support [11,12]. Nevertheless, studies on CPCs have faced a common drawback: a slow resorption rate that inherently compromises new bone ingrowth [13,14]. To address these challenges, researchers have explored various strategies to accelerate the remodeling capacity of adhesive bone cements. Specifically, increasing material porosity to mimic physiological bone architecture has been proposed to enhance resorption rate while sustaining coordinated bone remodeling [15,16].

Carbonates have demonstrated substantial potential for enhancing the properties of bone cements due to their natural compatibility with bone mineral content and their ability to promote osteogenesis [17,18]. Moreover, harnessing the chemical properties of carbonates to drive CO_2_ release offers a straightforward method for generating controlled porosity within the material matrix, exponentially increasing its specific surface area and permeability. This porous architecture provides vital interconnected channels for cell migration, nutrient diffusion, and angiogenesis, effectively accelerating the degradation and remodeling of the biomaterial [19]. Furthermore, a porous structure mimics the microenvironment of natural cancellous bone, facilitating osteoblast attachment and proliferation to accelerate *de novo* bone formation [20].

While Sodium Carbonate (Na_2_CO_3_) has traditionally been utilized in cement engineering as a hydration accelerator to enhance early-age structural strength, its utility extends well beyond industrial applications. Combining alkaline carbonates with gas-foaming technologies offers a highly compelling strategy for the fabrication of advanced tissue engineering scaffolds [21]. However, despite extensive research on calcium phosphate-based biomaterials and scaffolds for bone regeneration, the effects of carbonate-induced pore formation on the microstructure and biomechanical properties of CPC around dental implants are not yet fully understood. Consequently, the novelty of this study lies in using dry Na_2_CO_3_ as an in situ foaming agent within a CPC matrix for peri-implant bone grafting [22].

Therefore, we hypothesized that this gas-mediated pore-forming strategy would accelerate the material’s resorption rate while allowing us to evaluate the critical trade-off between porous architecture and maintaining mechanical integrity. To test this, we investigated how the addition of Na_2_CO_3_ affects the cement’s structural morphology and biomechanical performance.

## 2. Materials and Methods

### 2.1. Material Synthesis and Group Formulation

**Figure 1:**
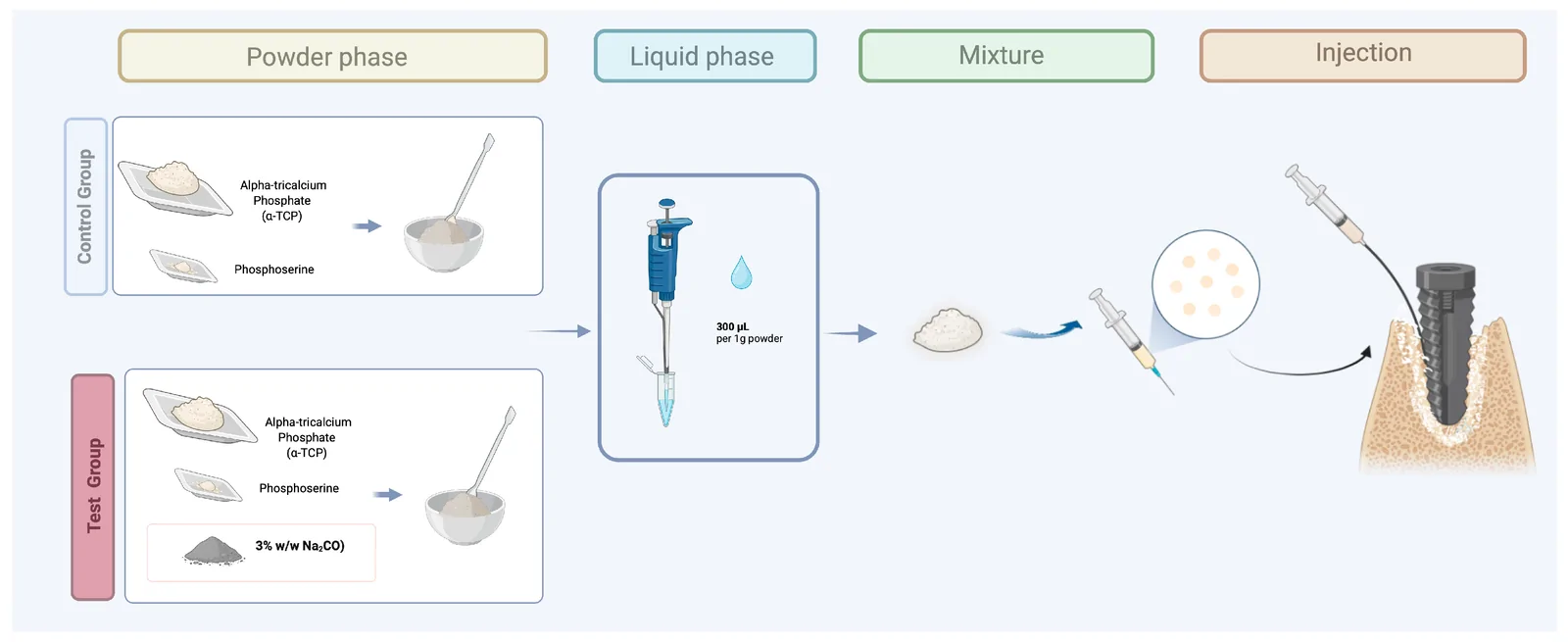
Formulation of the biomaterial groups. The baseline bone cement was composed of a 3:1 weight ratio of α-TCP and the amino acid phosphoserine, hydrated with ultrapure laboratory water to form the control group (CG). For pore creation, 3 wt.% of anhydrous sodium carbonate (Na_2_CO_3_) was incorporated into the powder blend and hydrated at a liquid-to-powder (L/P) ratio of 300 μL:1 g to form the test group (TG).

The baseline bone cement material (Control Group, CG) was formulated by mixing α-tricalcium phosphate (α-TCP) and phosphoserine in a 3:1 *w*/*w* ratio. Based on preliminary handling tests, 3 wt.% Na_2_CO_3_ (Merck, Darmstadt, Germany) was selected as the highest concentration that maintained acceptable injectability and was incorporated into the solid precursor powder to form the proof-of-concept test group (TG). Both groups were then hydrated with ultrapure laboratory water at a liquid-to-powder (L/P) ratio of 300 μL:1 g, selected based on preliminary handling tests to ensure adequate mixing and injectability (Table 1 and Figure 1).

Mixing was performed manually for ~10 s using a metal spatula. The bone cement pastes were then injected into different molds according to the purpose to cure and set completely for 24 h at 37 °C and 100% relative humidity to mimic simulated physiological conditions.

### 2.2. Morphological and Structural Characterization

#### 2.2.1. Scanning Electron Microscopy (SEM)

The morphology of the bone cements was evaluated using scanning electron microscopy (SEM). Dried samples were coated with a 4.0 nm platinum layer at 10^−7^ mbar using an EM ACE600 sputter coater (Leica Microsystems, Wetzlar, Germany) to enhance electrical conductivity and image resolution. SEM micrographs were captured at 15.00 kV in a vacuum of 1.25 × 10^−6^ mbar using a 30.00 μm aperture. Images were acquired at 25× and 500× magnification using a Zeiss Crossbeam 340 SEM (Carl Zeiss, Oberkochen, Germany).

#### 2.2.2. Micro-Computed Tomography (μCT)

Cylindrical samples (5 mm diameter) were scanned using a multiscale X-ray microscope (SKYSCAN 2214, Bruker μCT, Kontich, Belgium) at 60 kV and 140 µA, acquiring 3601 projections (3066 × 1944 px) at 0.1° intervals over 360°, with 1290 ms exposure. Images were reconstructed using NRecon software (v2.2.0.6) at 4.5 µm voxel resolution. Each dataset was cropped to 4 mm × 2.25 mm volumes of interest (VOIs) and further partitioned into seven sub-VOIs (1.12 mm × 2.25 mm) for porosity analysis using Otsu thresholding and 3D morphological processing (CTAn v1.23.0.2). 3D renderings were then generated with CTvox (Version 3.3.1, Bruker μCT, Kontich, Belgium).

### 2.3. Chemical Characterization

To characterize the chemical properties, Fourier transform infrared (FTIR) spectroscopy (Bruker Optik GmbH, Ettlingen, Germany) was performed. The spectrometer was equipped with a silicon carbide globar as the IR source and an internal deuterated L-alanine-doped triglycine sulfate (dLATGS) single detector operating at room temperature. The main spectrometer featured a diamond attenuated total reflectance (ATR) unit (Platinum ATR unit, Bruker Optik GmbH) for direct measurement. The computer-controlled stage facilitated the placement of a gold-coated mirror for reflectance measurements or the insertion of round calcium fluoride (CaF2) sample filter plates (Korth Kristalle GmbH, Kiel, Germany) for transmittance measurements. Absorbance spectra were collected over a range of 400–4000 cm^−1^. During spectral acquisition and analysis, the system was operated using the proprietary 32-bit OPUS software (v8.7, Bruker Optik GmbH).

### 2.4. Mechanical Characterization

#### 2.4.1. Removal Torque Test (RTT)

The removal torque apparatus included a custom-built environmental chamber maintained at 37 °C and 90% humidity to simulate the intraoral microenvironment. Testing was conducted in accordance with ISO 13498:2011 [23] using Roxolid, SLActive, and Tissue Level (TL; Ø 4.1 × 4 mm) implants (Institut Straumann AG, Basel, Switzerland) with a predefined cement gap width of 1.5 mm. Removal torque was measured at an angular velocity of 6°/s, with five implants per dataset. Implants were reused following a standardized cleaning protocol (10% citric acid, ultrasonic cleaning, and ultrapure water rinsing) to ensure reliability without affecting bench test results.

#### 2.4.2. Compression Modulus Test (CMT)

Cylindrical specimens were prepared using custom-made molds (4 mm diameter × 6 mm height). Twenty-four hours post-mixing, each specimen was positioned vertically within the central groove of the lower fixture, and a compressive load was applied along its longitudinal axis. Testing was executed utilizing a Universal Testing Machine (Zwick Roell Z1.0, Ulm, Germany) operated via testXpert^®^ Il V3.6. The maximum force at fracture was recorded to calculate the compressive strength using the following equation:

$$P=\frac{F}{\pi \times r^{2}}$$

where *P* represents the compressive strength in megapascals (MPa), *F* is the maximum recorded force at fracture in Newtons (N), and *r* is the radius of the cylindrical specimen (1.96 mm). Young’s modulus was determined from the slope of the linear region of the compressive stress–strain curve in accordance with ASTM D695 [24].

#### 2.4.3. Hardness Test (HT)

Hardness testing was conducted in accordance with ISO 48-4 [25]. Bone cement pastes were filled into plate-shaped molds, and excess material was scraped off to ensure parallel surfaces. After setting for 24 h at 23 ± 1 °C, the samples were demolded. Plane parallelism was verified and adjusted via grinding paper if necessary. A hardness testing machine (SCHMIDT, Passau, Germany) was used, with each sample measured at five different positions to determine the average hardness value.

### 2.5. Statistical Analysis

Statistical analysis was performed using GraphPad Prism 10.1.1. Results are expressed as mean ± standard deviation (SD). Statistical significance between the CG and the TG was analyzed using the Mann–Whitney test, with significance defined as *p* < 0.05.

## 3. Results

### 3.1. Morphological and Structural Properties

**Figure 2:**
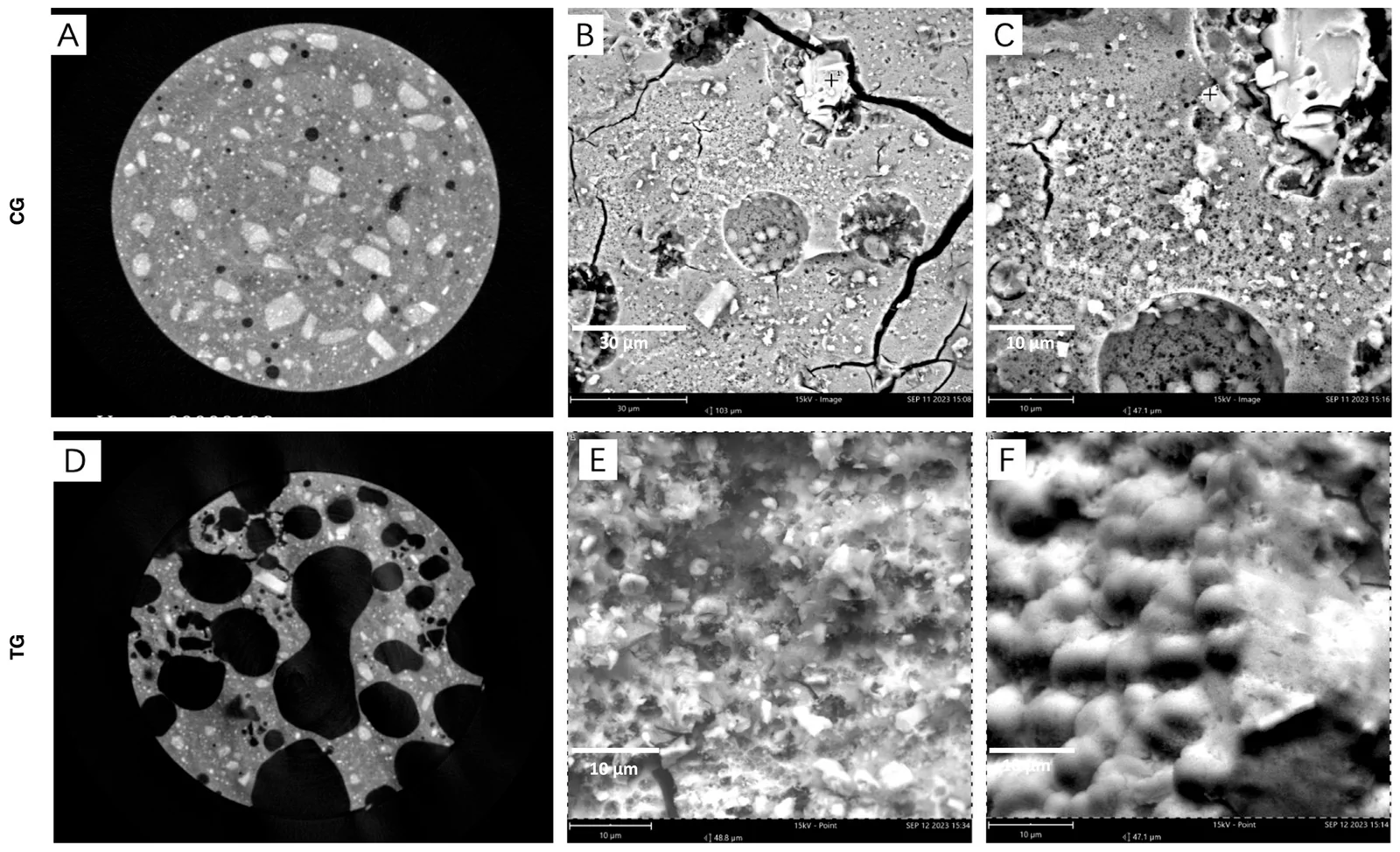
Structural and morphological characterization of bone cements after 24 h of setting. (**A**–**C**) CG: (**A**) 2D cross-sectional μ-CT image showing a dense, solid internal structure; (**B**,**C**) SEM micrographs at varying magnifications showing packed sheets of dense crystals with micro-fractures. (**D**–**F**) TG: (**D**) 2D cross-sectional μ-CT image showing the formation of large internal macropores and a foam-like structure; (**E**,**F**) SEM micrographs revealing a uniformly distributed, finely porous matrix composed of smaller plate-like crystals and nodular micro-textures.

To evaluate the internal architecture and surface topography of the cured bone cements, μ-CT and SEM characterizations were performed after 24 h of setting. Distinct morphological and structural variations were observed between the CG and the TG (Figure 2).

The 2D cross-sectional μ-CT slices revealed a highly dense and homogeneous internal structure for the CG, characterized by the tight packing of mineral aggregates with minimal intrinsic voids (Figure 2A). In contrast, the μ-CT image of the TG exhibited interconnected macroscopic voids and a porous network embedded within the bone cement matrix (Figure 2D). This demonstrated that the incorporation of 3 wt.% Na_2_CO_3_ successfully induced in situ gas foaming during the initial hydration process.

SEM also highlighted the corresponding alterations in the microcrystalline matrix. At the microscale, the CG displayed a highly consolidated, dense microstructure consisting of tightly packed, continuous sheets of large crystals, with substantial microcracks throughout the matrix (Figure 2B,C). Conversely, the SEM micrographs of the TG revealed a clearly different microstructural morphology. The matrix in TG was composed of uniformly distributed, smaller plate-like structures, forming a highly continuous, finely textured porous crystalline network (Figure 2E,F).

### 3.2. Chemical Characterization

**Figure 3:**
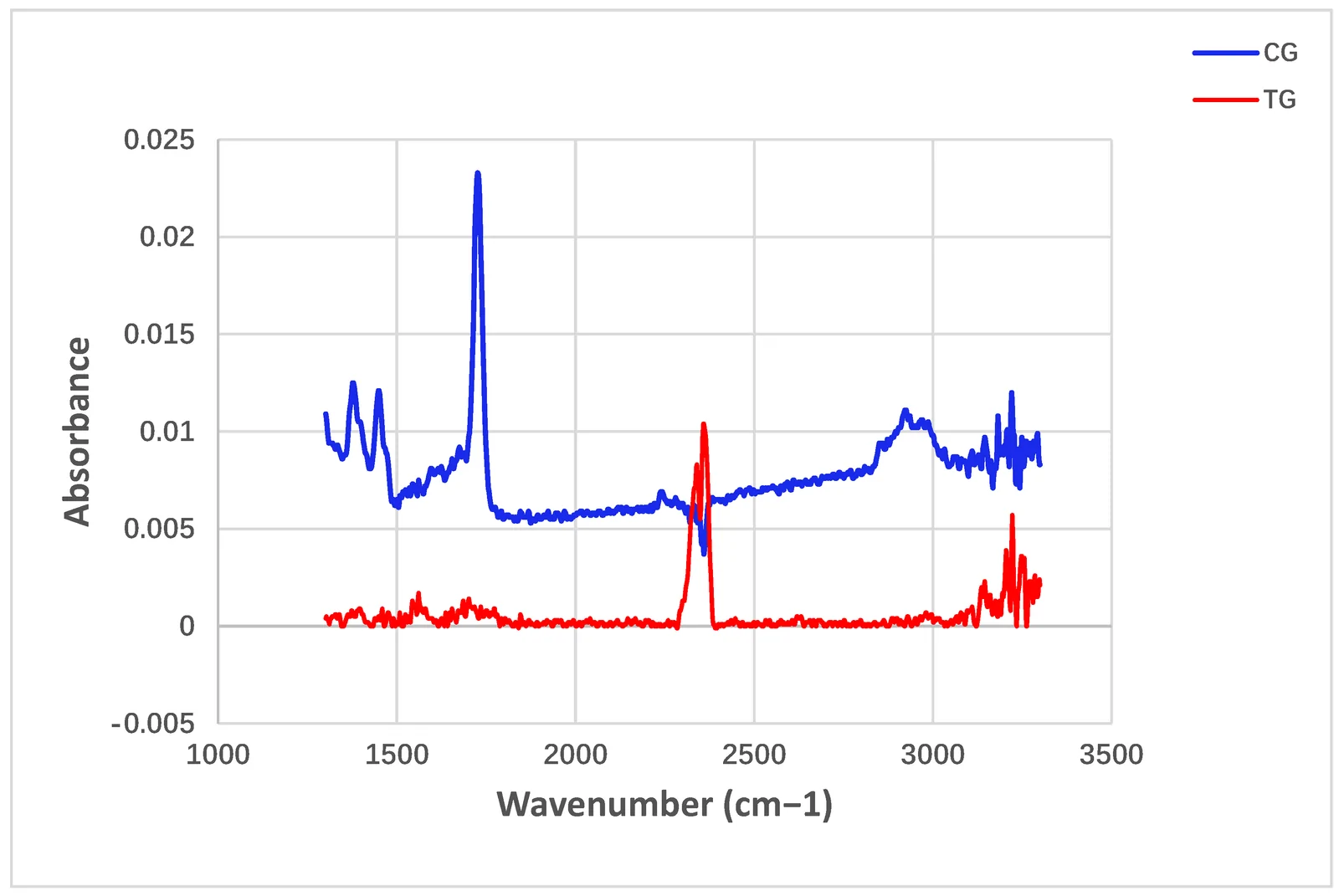
FTIR spectra of CG and TG after 24 h of setting. The CG (blue) shows the characteristic peak of phosphoserine at ~1730. The TG (red) features a distinct gas peak at ~2349, confirming successful in situ gas generation and structural entrapment.

FTIR spectra (Figure 3) revealed changes in the chemical structure after 24 h of setting. For the CG (blue curve), absorption peaks in the 1500–1750 cm^−1^ range confirmed the presence of carbonyl groups (C=O). The sharp peak at approximately 1730 cm^−1^ is assigned to the C=O stretching vibration associated with phosphoserine. In contrast, the TG (red curve) exhibited a prominent new peak at around 2349 cm^−1^, which is the definitive signature of molecular gas. This directly supports that the addition of Na_2_CO_3_ successfully triggered an in situ gas-foaming reaction during hydration.

### 3.3. Mechanical Properties

#### 3.3.1. Removal Torque Analysis

**Figure 4:**
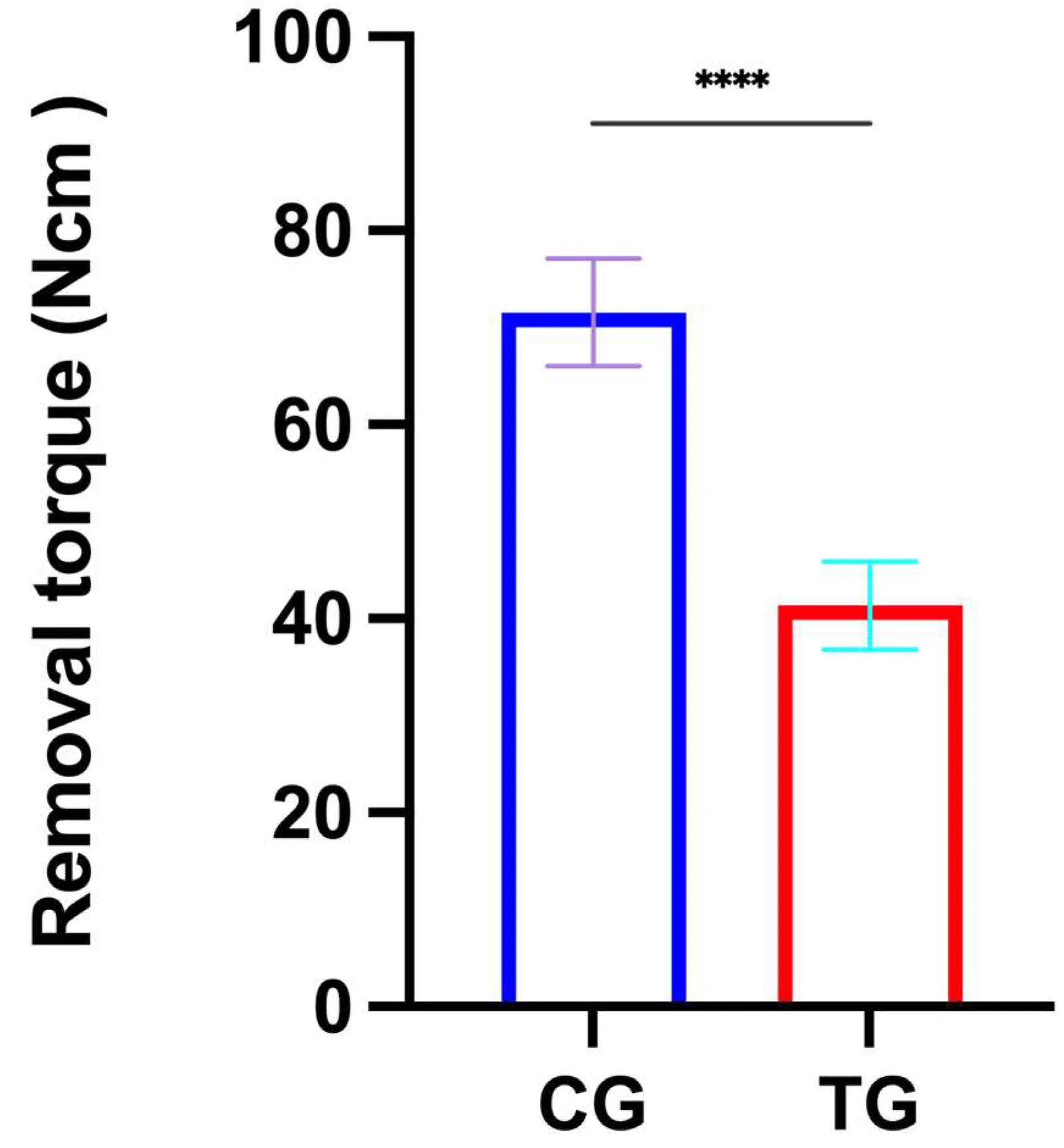
Removal torque values for the implants anchored with CG and TG bone cements. Bars represent the mean ± SD, with individual data points plotted. **** = *p* < 0.0001.

The primary stability of the implant and the adhesive performance of the bone cements were evaluated using the removal torque test (Figure 4). The CG demonstrated a significantly higher mean removal torque (71.58 ± 5.56 N/cm) than the TG (41.37 ± 4.54 N/cm) (*p* < 0.0001). This indicates that the non-porous baseline material provides superior initial adhesion and torsional stability for simulated implants in situ.

#### 3.3.2. Compression Modulus Results

**Figure 5:**
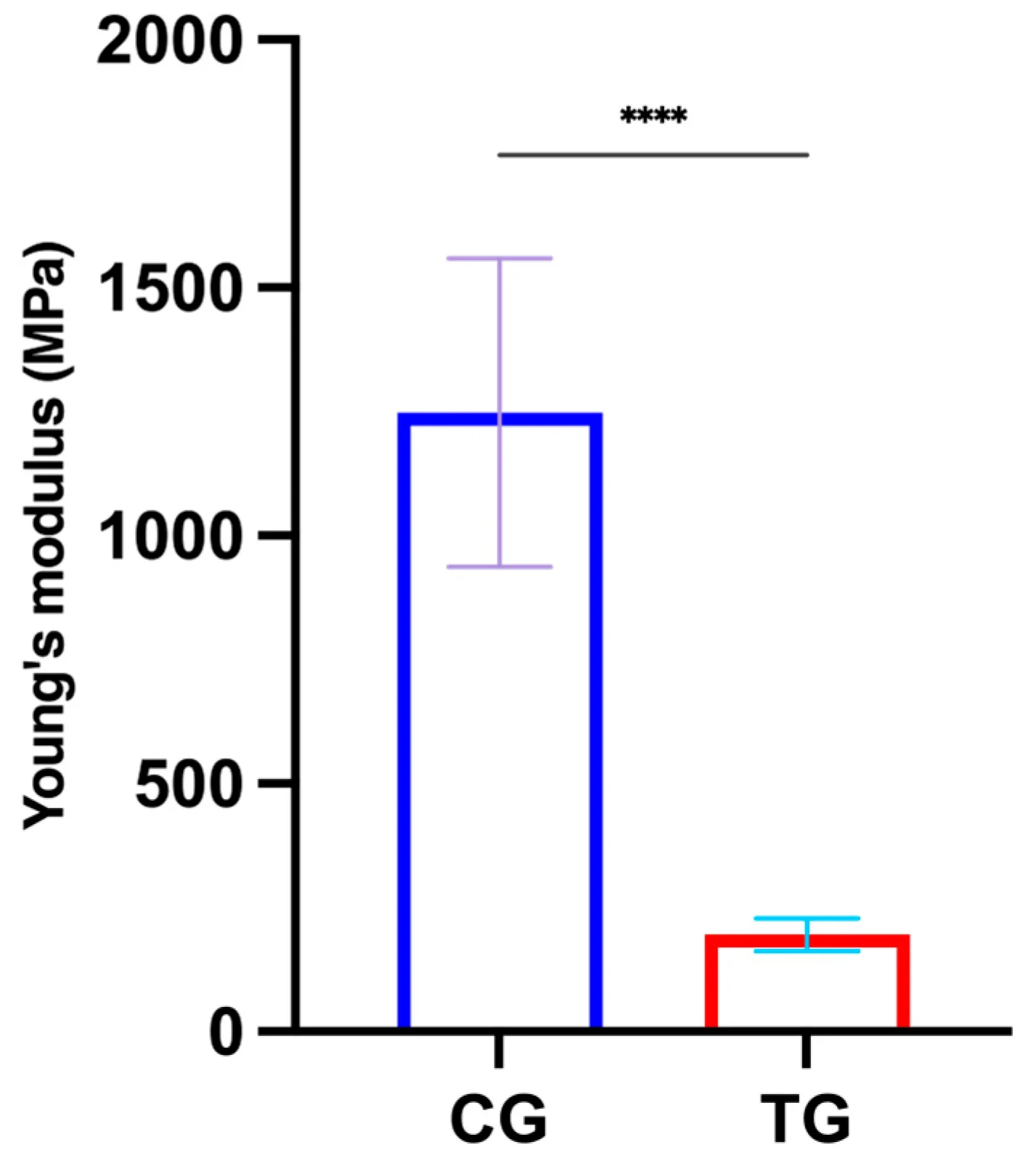
Young’s modulus of the CG and TG bone cements. Bars represent the mean ± SD with individual data points plotted. **** = *p* < 0.0001.

The mechanical stiffness of the cured bone cements was evaluated via compression testing to determine their Young’s modulus (Figure 5). The CG exhibited a substantially higher Young’s modulus (1248.02 ± 278.21 MPa) compared to the TG, which dropped to 195.42 ± 29.55 MPa (*p* < 0.0001). This sharp reduction in stiffness clearly demonstrates that, while in situ gas generation successfully introduces the desired porous architecture, the resulting internal voids severely compromise the structural integrity and load-bearing capacity of bone cements.

#### 3.3.3. Hardness Results

**Figure 6:**
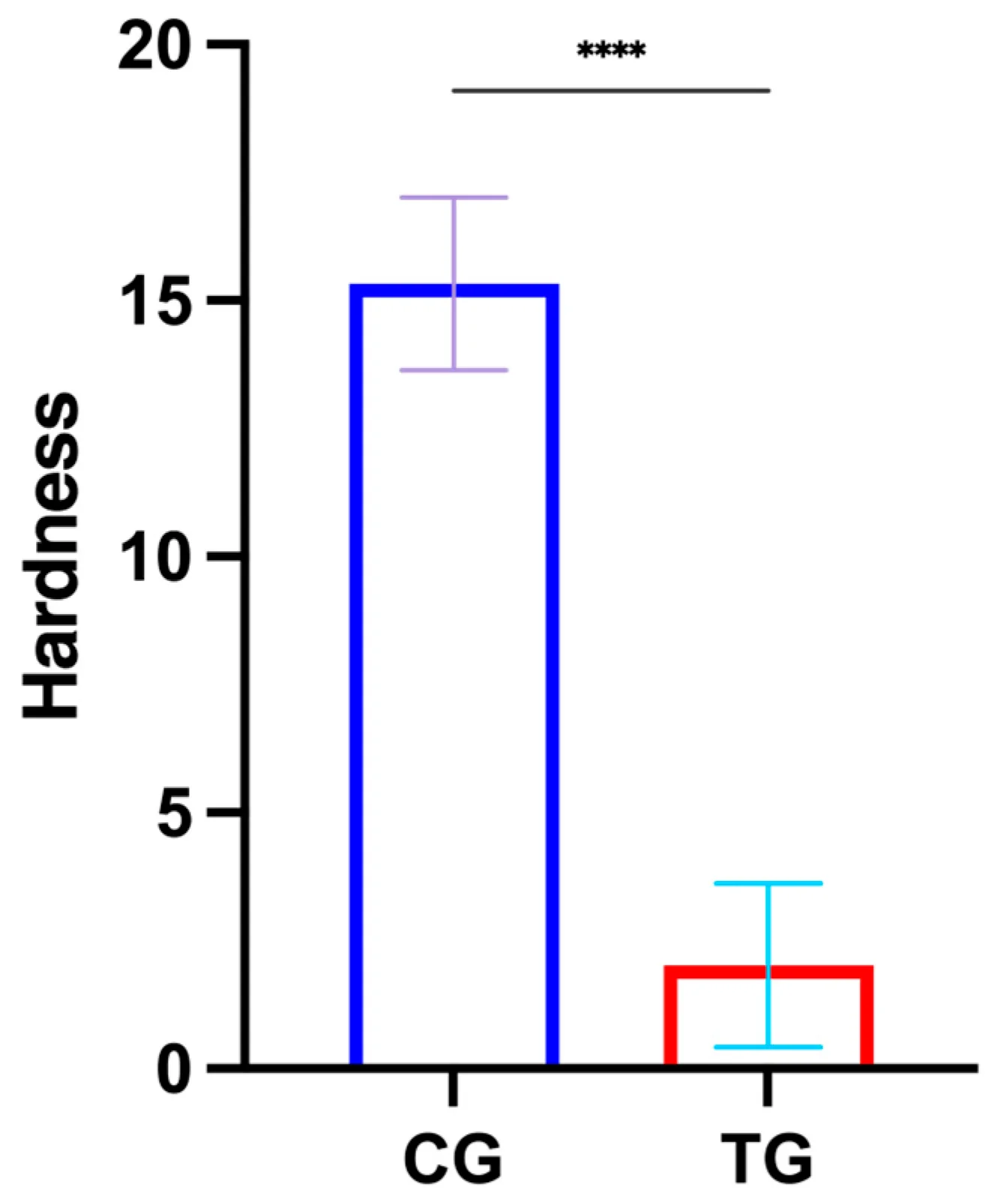
Surface hardness of the CG and TG bone cements. Bars represent the mean ± SD with individual data points plotted. **** = *p* < 0.0001.

Surface hardness testing showed a trend consistent with the other mechanical metrics (Figure 6). The CG maintained a stable surface hardness of 15.31 ± 1.63, which dropped significantly to 2.01 ± 1.58 in the TG (*p* < 0.0001). This severe loss in hardness indicates that the introduction of the porous network significantly compromises the material’s resistance to localized surface deformation, making the set bone cement considerably more brittle and fragile under concentrated loads.

## 4. Discussion

The present study confirmed the effective creation of pores in the CPC matrix by adding carbonates via a gas-released chemical reaction. As expected, increased material porosity negatively affected the biomechanical strength of the CPC, as evidenced by significantly lower removal torque, compression, and hardness test values.

In the formulation of CPC-based bone cements, α-TCP is frequently selected as the main precursor for CPC synthesis due to its high solubility and thermodynamic instability in aqueous solutions [26,27]. Upon hydration, α-TCP rapidly hydrolyzes and precipitates into calcium-deficient hydroxyapatite (CDHA), an isomorphic phase that closely mimics the biological composition of native bone while facilitating the hardening of the cement matrix [28]. Bone cements are currently in the spotlight of implant research, with a focus on developing biomaterials for peri-implant bone filling [29,30]. These materials aim to provide intimate contact with the implant surface for optimal stability while offering a favorable biological environment for bone healing/regeneration [31,32]. Additionally, the material’s liquid consistency increases the number of indications, as its use is not limited to larger defects (as with most particulate materials); it can also be used for narrow defects and even after implant placement to reinforce the osseointegration of implants with compromised primary stability [33]. As previously mentioned, the major limitation of CPC is its low resorption rate that directly interferes with the bone formation phase.

The primary objective of this study was to generate porosity in the basic CPC to improve its slow resorption rate. The incorporation of Na_2_CO_3_ successfully initiated a chemical reaction upon hydration. Pilot screening identified 3 wt.% as the highest concentration that maintained injectability, as confirmed by FTIR analysis, which demonstrated distinct CO_2_ peaks. Respective gas generation resulted in an altered, crystalline-porous microstructure, as observed in μ-CT and SEM analyses. Although the μCT and SEM images showed pore formation, they did not provide a detailed quantitative characterization of the porous structure. Further studies should assess total porosity, pore-size distribution, pore connectivity, and spatial variation.

While porosity is biologically favorable for osteoconduction, cellular infiltration, and angiogenesis, it is negatively correlated to biomechanical properties of the material [34], and study outcomes underscore this well-documented engineering trade-off in bioceramics. TG exhibited a severe reduction across all mechanical parameters: removal torque, compressive modulus, and hardness. In a previous study using a comparable adhesive cement–implant system, a removal torque of >32 N/cm was suggested as a benchmark for further material development [35]. Although the TG showed a significantly lower removal torque than the CG, its mean value of 41.37 ± 4.54 N/cm remained above this threshold. The expansion of gas created internal voids that acted as stress concentrators, compromising the structural integrity of the set bone cement and rendering the material significantly more brittle. Compared with the existing literature [36], it is consistently observed that as total porosity increases, the compressive strength of CPCs decreases exponentially. Therefore, while the Na_2_CO_3_-modified bone cement may offer superior biological remodeling potential, its current mechanical profile limits its application in load-bearing clinical scenarios.

Limitations of this study should also be noted. First, this study serves primarily as a proof-of-concept, highlighting the critical importance of achieving a better balance between biological porosity and mechanical stability. Second, the evaluation was limited to a single test group with a fixed concentration of Na_2_CO_3_; therefore, the present study does not establish 3 wt.% as the optimal formulation. Additionally, crystallographic phase characterization via powder X-ray diffraction (XRD) was not performed, leaving the precise impact of carbonate addition on the cement’s crystal structure and phase composition after hydration to be determined.

To address these limitations, ongoing and future research should investigate alternative foaming agents (e.g., sodium bicarbonate or magnesium carbonate) and optimize formulation ratios [37,38,39] and incorporate XRD analyses to fully map the phase transformation kinetics. Once a balanced candidate formulation is identified, comprehensive in vitro biocompatibility assays and subsequent preclinical in vivo studies should be performed to validate its clinical efficacy in bone defects.

## 5. Conclusions

The incorporation of 3 wt.% carbonate salts (Na_2_CO_3_) into a calcium phosphate/phosphoserine bone cement successfully acted as a porogen by generating CO_2_ gas in situ immediately upon hydration, as demonstrated by μCT and SEM analyses. Despite a substantial increase in bone cement porosity, this protocol negatively affected the material’s biomechanical properties. Compared with the control group, the Na_2_CO_3_-modified TG exhibited a significant decrease in removal torque from 71.58 ± 5.56 to 41.37 ± 4.54 N/cm, Young’s modulus from 1248.02 ± 278.21 to 195.42 ± 29.55 MPa, and surface hardness from 15.31 ± 1.63 to 2.01 ± 1.58 (*p* < 0.0001 for all comparisons). These findings demonstrate a clear trade-off between the generation of a porous architecture and the mechanical integrity of bone cement. Further optimization is necessary to balance the required porosity and mechanical strength. Once an optimized formulation is established, comprehensive in vitro biocompatibility assessments, including cytotoxicity testing, followed by preclinical in vivo evaluation, will be necessary to assess its biological safety and regenerative potential for peri-implant applications.

## Figures and Tables

**Table 1:** Group formulation.

Control Group (CG)	Consisted of the baseline α-TCP and phosphoserine powder blend. Hydration was initiated by adding ultrapure laboratory water to a liquid-to-powder (L/P) ratio of 300 μL:1 g.
Test Group (TG)	Consisted of the baseline powder blend, dry-blended with 3 wt.% of anhydrous sodium carbonate (Na_2_CO_3_, Merck, Germany). Hydration was conducted using the identical L/P ratio of 300 μL:1 g with ultrapure laboratory water.

## Data Availability

The data used to support the findings of this study are available from the corresponding authors upon reasonable request.

## References

[1] Javed F., Ahmed H.B., Crespi R., Romanos G.E. (2013). Role of primary stability for successful osseointegration of dental implants: Factors of influence and evaluation. Interv. Med. Appl. Sci..

[2] Lioubavina-Hack N., Lang N.P., Karring T. (2006). Significance of primary stability for osseointegration of dental implants. Clin. Oral Implants Res..

[3] Pandey C., Rokaya D., Bhattarai B.P. (2022). Contemporary Concepts in Osseointegration of Dental Implants: A Review. Biomed. Res. Int..

[4] Zhao R., Yang R., Cooper P.R., Khurshid Z., Shavandi A., Ratnayake J. (2021). Bone Grafts and Substitutes in Dentistry: A Review of Current Trends and Developments. Molecules.

[5] Kittur N., Oak R., Dekate D., Jadhav S., Dhatrak P. (2021). Dental implant stability and its measurements to improve osseointegration at the bone-implant interface: A review. Mater. Today Proc..

[6] Sculean A., Nikolidakis D., Nikou G., Ivanovic A., Chapple I.L., Stavropoulos A. (2015). Biomaterials for promoting periodontal regeneration in human intrabony defects: A systematic review. Periodontol. 2000.

[7] Sanz M., Dahlin C., Apatzidou D., Artzi Z., Bozic D., Calciolari E., De Bruyn H., Dommisch H., Donos N., Eickholz P. (2019). Biomaterials and regenerative technologies used in bone regeneration in the craniomaxillofacial region: Consensus report of group 2 of the 15th European Workshop on Periodontology on Bone Regeneration. J. Clin. Periodontol..

[8] You L., Temiyasathit S., Lee P., Kim C.H., Tummala P., Yao W., Kingery W., Malone A.M., Kwon R.Y., Jacobs C.R. (2008). Osteocytes as mechanosensors in the inhibition of bone resorption due to mechanical loading. Bone.

[9] Epsley S., Tadros S., Farid A., Kargilis D., Mehta S., Rajapakse C.S. (2020). The Effect of Inflammation on Bone. Front. Physiol..

[10] Matos G.R.M. (2021). Surface Roughness of Dental Implant and Osseointegration. J. Maxillofac. Oral Surg..

[11] Renner T., Otto P., Kübler A.C., Hölscher-Doht S., Gbureck U. (2023). Novel adhesive mineral-organic bone cements based on phosphoserine and magnesium phosphates or oxides. J. Mater. Sci. Mater. Med..

[12] Bojan A.J., Stadelmann V.A., Wu D., Pujari-Palmer M., Insley G., Sundh D., Persson C., Engqvist H., Procter P. (2022). A new bone adhesive candidate- does it work in human bone? An ex-vivo preclinical evaluation in fresh human osteoporotic femoral head bone. Injury.

[13] Combes C., Miao B., Bareille R., Rey C. (2006). Preparation, physical-chemical characterisation and cytocompatibility of calcium carbonate cements. Biomaterials.

[14] Mirtchi A.A., Lemaitre J., Terao N. (1989). Calcium phosphate cements: Study of the β-tricalcium phosphate—monocalcium phosphate system. Biomaterials.

[15] del Real R.P., Wolke J.G., Vallet-Regí M., Jansen J.A. (2002). A new method to produce macropores in calcium phosphate cements. Biomaterials.

[16] Cinici B., Yaba S., Kurt M., Yalcin H.C., Duta L., Gunduz O. (2024). Fabrication Strategies for Bioceramic Scaffolds in Bone Tissue Engineering with Generative Design Applications. Biomimetics.

[17] Bian Y., Duan X., Han S., Yuan Y., Shen J., Wang Z., Han B. (2026). Application and Research Progress of Carbonated Hydroxyapatite in Bone Tissue Regeneration. Adv. Biol..

[18] Li X., Yang X., Liu X., He W., Huang Q., Li S., Feng Q. (2018). Calcium carbonate nanoparticles promote osteogenesis compared to adipogenesis in human bone-marrow mesenchymal stem cells. Prog. Nat. Sci. Mater. Int..

[19] Elsheikh M., Kishida R., Hayashi K., Tsuchiya A., Shimabukuro M., Ishikawa K. (2022). Effects of pore interconnectivity on bone regeneration in carbonate apatite blocks. Regen. Biomater..

[20] Shah S.A., Sohail M., Nakielski P., Rinoldi C., Zargarian S.S., Kosik-Kozioł A., Ziai Y., Haghighat Bayan M.A., Zakrzewska A., Rybak D. (2025). Integrating Micro- and Nanostructured Platforms and Biological Drugs to Enhance Biomaterial-Based Bone Regeneration Strategies. Biomacromolecules.

[21] Skalny J., Phillips J.C., Cahn D.S. (1973). Low water to cement ratio concretes. Cem. Concr. Res..

[22] Andrada A.S., Maria D.A., da Silva S.N., Santos L., Jansen J.A. (2025). Calcium Phosphate Cements: From the 1980s to the 2020s. Tissue Eng. Part C Methods.

[23] (2011). Dentistry—Torsion Test of Implant Body/Connecting Part Joints of Endosseous Dental Implant Systems.

[24] (2023). Standard Test Method for Compressive Properties of Rigid Plastics.

[25] (2018). Rubber, Vulcanized or Thermoplastic—Determination of Hardness—Part 4: Indentation Hardness by Durometer Method (Shore Hardness).

[26] Tronco M.C., Cassel J.B., Dos Santos L.A. (2022). α-TCP-based calcium phosphate cements: A critical review. Acta Biomater..

[27] Carrodeguas R.G., De Aza S. (2011). α-Tricalcium phosphate: Synthesis, properties and biomedical applications. Acta Biomater..

[28] Du W., Guo Y., Pathak J.L., Xiaoshi C., Su H., Wang L. (2026). Harnessing and Optimizing α-TCP for Oral Tissue Engineering and Regenerative Dentistry. Int. Dent. J..

[29] Schickert S.L., Jansen J.A., Bronkhorst E.M., van den Beucken J.J., Leeuwenburgh S.C. (2020). Stabilizing dental implants with a fiber-reinforced calcium phosphate cement: An in vitro and in vivo study. Acta Biomater..

[30] Brochu B.M., Sturm S.R., Kawase De Queiroz Goncalves J.A., Mirsky N.A., Sandino A.I., Panthaki K.Z., Panthaki K.Z., Nayak V.V., Daunert S., Witek L. (2024). Advances in Bioceramics for Bone Regeneration: A Narrative Review. Biomimetics.

[31] Ahn Y., Desai P.K., Rainey A., Ayala-Ortiz J.L., Ponnazhagan S., Gilbert S.R., Jun H.W., Cheon K. (2026). Innovations in Implant Osseointegration: Biomaterials, Surface Engineering, and Translational Strategies. J. Biomed. Mater. Res. A.

[32] Sorca B.V., Kaya D.A., Kaya M.G.A., Enachescu M., Ghetu D.M., Enache L.B., Boerasu I., Coman A.E., Rusu L.C., Constantinescu R. (2025). Bone Fillers with Balance Between Biocompatibility and Antimicrobial Properties. Biomimetics.

[33] O’Neill R., McCarthy H.O., Montufar E.B., Ginebra M.P., Wilson D.I., Lennon A., Dunne N. (2017). Critical review: Injectability of calcium phosphate pastes and cements. Acta Biomater..

[34] Khajehmohammadi M., Azizi Tafti R., Nikukar H. (2023). Effect of porosity on mechanical and biological properties of bioprinted scaffolds. J. Biomed. Mater. Res. Part A.

[35] Andersen O.Z., Bellon B., Lamkaouchi M., Brunelli M., Wei Q., Procter P., Pippenger B.E. (2023). Determining primary stability for adhesively stabilized dental implants. Clin. Oral Investig..

[36] Xu H.H., Burguera E.F., Carey L.E. (2007). Strong, macroporous, and in situ-setting calcium phosphate cement-layered structures. Biomaterials.

[37] Wang S., Xu C., Yu S., Wu X., Jie Z., Dai H. (2019). Citric acid enhances the physical properties, cytocompatibility and osteogenesis of magnesium calcium phosphate cement. J. Mech. Behav. Biomed. Mater..

[38] Jia J., Zhou H., Wei J., Jiang X., Hua H., Chen F., Wei S., Shin J.W., Liu C. (2010). Development of magnesium calcium phosphate biocement for bone regeneration. J. R. Soc. Interface.

[39] Sheu M.T., Huang J.C., Yeh G.C., Ho H.O. (2001). Characterization of collagen gel solutions and collagen matrices for cell culture. Biomaterials.

